# Questionnaire-based approach to evaluate the convenience of rechargeable extracorporeal pulse generators for wireless spinal cord stimulation

**DOI:** 10.1038/s41598-022-11778-5

**Published:** 2022-05-17

**Authors:** Mohammad Mehdi Hajiabadi, Martin Jakobs, Petya Vicheva, Andreas Unterberg, Rezvan Ahmadi

**Affiliations:** 1 Department of Neurosurgery, University Hospital Heidelberg, Im Neuenheimer Feld 400, 69120 Heidelberg, Germany; 2 Division of Surgical Pain Management, Department of Neurosurgery, University Hospital Heidelberg, Heidelberg, Germany

**Keywords:** Neuropathic pain, Peripheral neuropathies, Spinal cord diseases

## Abstract

Spinal cord stimulation (SCS) has been utilized for more than 50 years to treat refractory neuropathic pain. Currently, SCS systems with fully implantable pulse generators (IPGs) represent the standard. New wireless extracorporeal SCS (wSCS) devices without IPGs promise higher levels of comfort and convenience for patients. However, to date there are no studies on how charging and using this wSCS system affects patients and their therapy. This study is the first questionnaire-based survey on this topic focusing on patient experience. The trial was a single arm, open-label and mono-centric phase IV study. Standardized questionnaires were sent to all patients with a wSCS device in use at the time of trial. The primary endpoint was the convenience of the charging and wearing process scored on an ordinal scale from "very hard" (1) to "very easy" (5). Secondary endpoints included time needed for charging, the duration of stimulation per day and complication rates. Questionnaires of 6 out of 9 patients were returned and eligible for data analysis. The mean age of patients was 61.3 ± 6.7 (± SD) years. The duration of therapy was 20.3 ± 15.9 months (mean ± SD). The mean duration of daily stimulation was 17 ± 5.9 h (mean ± SD). n = 5 patients rated the overall convenience as "easy" (4) and n = 3 patients evaluated the effort of the charging process and wearing of the wSCS device as "low" (4). n = 5 patients considered the wearing and charging process as active participation in their therapy. n = 5 patients would choose an extracorporeal device again over a conventional SCS system. Early or late surgical complications did not occur in this patient collective. Overall, patients felt confident using extracorporeal wSCS devices without any complications. Effort to maintain therapy with this system was rated as low.

## Introduction

Spinal cord stimulation (SCS) is an established method for the treatment of neuropathic pain syndromes. Since the first application more than 50 years ago, the technology has been continuously improved^1,2^. Currently, conventional systems consist of two components: a pulse generator (PG), which acts as a power source and one or two leads (percutaneous or paddle) that are implanted in the spinal epidural space and transmit stimulation to the dorsal columns of the spinal cord^3^. The first fully implantable pulse generators were non-rechargeable. As primary cell battery systems, they have fixed capacities that need repetitive surgical procedures every 2–5 years to the replace the device once the capacity has been fully exploited^4,5^.

To allow for lifelong therapy without compromising on stimulation parameters and to reduce complications of repetitive replacement surgeries, further innovations were developed^4,6–8^. In 2004 the first rechargeable IPG with a projected lifespan of 15 or more years—according to the manufactures—was introduced^1^. This is supposed to result in fewer replacements, thus reducing both the morbidity associated with surgical interventions as well as potential costs^6^. Since 2013, a wireless SCS (wSCS) system, which is powered by external pulse generators (EPGs) and requires percutaneous implantation of electrodes, that have a distal stimulation receiving part to be stored subcutaneously after anchoring the leas to the fascia, is available. The wireless SCS system eliminates the need to implant the IPG and the issue of pocket pain^9^. Once the battery capacity is exhausted, the EPGs must be replaced with another already charged one. This provides a permanent stimulation without relevant interruption of the therapy if the patients manage the charging and changing of the EPGs well. The recharging and wearing process therefore requires therapy awareness and certain cognitive and motor skills that may be impaired in patients of advanced age^10^.

To date, there are no guidelines for which type of pulse generator, internal or external, rechargeable or non-rechargeable, should be implanted in which patient for SCS therapy. In addition, it is still unknown whether the age of the patient, the underlying disease, the number of epidural electrodes or the type of stimulation may play important roles in deciding which SCS system to best use^1^.

The main objective of this study was to rate the convenience of wearing and recharging, user confidence and satisfaction (general satisfaction with the system beyond the charging process) with a wSCS system from the patients’ perspectives. Furthermore, this study focused on the following aspects: firstly, assessment of the burden of recharging; and secondly, documentation of long-term complications such as trouble with wearing the EPG, interruptions in therapy and failed recharges.

## Material and methods

The trial was a single arm, open-label and mono-centric phase IV study.

### Data collection

Our patient database was analyzed for eligible patients. Inclusion criteria were (1) Age 18 years and older; (2) Wireless SCS Implantation (Stimwave Freedom System, Stimwave Inc, USA); All patients were discussed in a multidisciplinary board perioperatively including an assessment for severe psychological comorbidities. (3) SCS therapy for at least 3 months at the time of the survey.

### Design and procedure

A study kit including general study information, a declaration of consent, the study questionnaire and a return envelope were sent to patients fulfilling the inclusion criteria. Completed questionnaires, which were returned within 6 weeks were included in data analysis. Informed consent to participate was obtained from all the participants. The questionnaire results were pseudonymized, coded and digitalized in a study database. The study database was supplemented with the following patient data from hospital charts: indication for SCS therapy, previous experiences with other SCS devices, date of surgery and surgical revisions.

### Questionnaire

The questionnaire contained 36 questions relating to relevant aspects of the wSCS device. 7 questions were open, and 29 questions were closed with 2 or more options, from which only the most suitable one could be chosen. 17 questions could only be answered with “yes” or “no”. Convenience of stimulation, wearing and recharging of EPG was rated on an ordinal scale from 1 to 5 (1 = very hard, 2 = hard, 3 = neutral, 4 = easy, 5 = very easy).

With regards to psychological influences this type of therapy may have on patients, the rate of agreement or disagreement could be given on an ordinal scale (“complete agreement”, “agreement”, “neutral”, “disagreement”, “complete disagreement”). Through the questionnaires, the patients were asked about their living conditions after implantation with the device (driving, pursuing a job and traveling), and whether the patients were already using with rechargeable electronic devices such as smart phones or tablets.

### Outcome measures

The primary endpoint of this trial was the evaluation of the convenience of wearing and recharging of the wSCS device. Secondary endpoints included the level of user confidence and satisfaction (general satisfaction with the system beyond the charging process), user acceptance of the wireless device, charge burden (defined as the time patients need to invest per week in charging the EPGs) and complication rates (e.g., surgical revisions, interruptions of stimulation due to disconnection between stimulator and receiver). In addition, patients were asked about the stimulator decision, desired shelf life, and important aspects of therapy.

### Statistical analysis

For continuous variables (e.g., age, duration of recharging and duration of stimulation) descriptive statistics (mean, standard deviation, range) were performed. The median was calculated when testing on an ordinal scale (convenience of recharging). The Shapiro–Wilk test was conducted to verify normal distribution. Statistical analysis was performed with SPSS (25 IBM corp., Armonk, New York, USA).

### Ethical considerations

This study was approved by the Ethics Committee of the University Hospital of Heidelberg. The principles of the Helsinki Declaration were followed.

## Results

### Patient characteristics

9 patients fulfilled the inclusion criteria and were sent a study kit. Out of these 6 (67%) questionnaires were returned and were deemed eligible for data analysis. The other 3 patients could not be reached by telephone either. 5 out of 6 patients were male (83%). n = 4 patients (66.6%) suffered from Failed-Back-Surgery-Syndrome (FBSS) as the most common diagnosis in our study cohort. n = 1 patient (16.7%) suffered from chronic painful polyneuropathy and n = 1 (16.7%) patient from chronic singultus. The mean age at the time of wSCS device implantation was 59.8 ± 6.3 years (range 50–66). 20.3 ± 15.9 months (range 8.9–43.9) was the average duration of therapy with the wSCS at the time of the survey. In contrast to age (*p* = 0.4), the length of therapy (*p* = 0.01) was not normally distributed according to the Shapiro–Wilk test.

### User introduction and user confidence

Patients received either 1–2 training sessions in handling and recharging of the device. In 83% (n = 5) of patients, a single training session with the device was sufficient to feel confident using the device. All patients felt adequately prepared to use the wSCS device after the last training session. At the time of study one patient did not feel confident with the wireless device at the time of the survey. 83% (n = 5) of patients reported a confident handling with the wireless devices after an average period of 2.2 weeks.

### Life with a wireless SCS device

The mean duration of stimulation per day was 17 ± 5.9 (range 10–24) hours. Only n = 2 (33.3%) patients did use the wSCS device overnight. 83% (n = 5) of patients drive a car. Two patients have travelled after surgery. Most patients, 83% (n = 5), regularly use a rechargeable electronic device such as smartphone or tablets.

### Recharging and wearing process

50% (n = 3) of patients checked the battery status of the wSCS device every 6 to 12 h. This checking interval varied between 15 min and 3 h for the rest of the patients (n = 3). 66% (n = 4) of the patients changed the EPG only when a warning signal was detected. n = 1 patient changed the stimulator when the battery status was less than 25% and n = 1 patient when the remaining battery capacity was between 50 and 75%. The mean duration of EPG charging was 2.25 ± 1.08 h (range 1–4). On average, patients rated the convenience of checking the battery status as “neutral”. Putting on and taking off the EPG was evaluated in n = 5 patient as “easy” to “very easy”. n = 1 patient evaluated putting on and taking off the EPG as “difficult”. Recharging the EPG was rated on average as “very easy”. Keeping the connection between the transmitter and implanted receiver during stimulation was evaluated as “very easy” by n = 5 patients and “easy” by n = 1 patient. The overall handling and EPG utilization were rated as “very easy” in n = 5 patients and “easy” in n = 1 patient. The overall effort was rated as “neutral” in n = 3 patients and “low” in n = 3 patients.

### User satisfaction

All patients (n = 6) were overall satisfied with the time required to recharge the EPG and checking the battery status. 83% (n = 5) of patients would recommend the system. N = 5 patients would choose EPG again if they had to decide between an IPG and EPG.

### Psychological aspects of wireless SCS therapy

The rating of the statement “Wearing the stimulator reminds me of my disease” was normally distributed. 2 patients reported this as an “agreement” 2 patients as “neutral” and 2 patients as “complete disagreement”. n = 5 patients agreed with the statement “wearing of EPG is an active participation in own treatment”. n = 5 patients completely disagreed with the allegation “fear of forgetting to recharge”.

### Complications

None of the patients had to undergo a surgical revision. Only one patient experienced more frequent interruptions between the transmitter and the micro-receiver. In n = 2 patients (33.3%), both stimulator and transmitter had to be changed due to hardware failures.

In the short form we present patient characteristics, duration of therapy and patient experience with therapy (Appendix 1). Additional data is available in a supplementary table (Appendix 2).

## Discussion

This study is the first trial in which convenience, patient satisfaction, charge burden with a wSCS system were examined. Our results suggest that the recharging and wearing process is overall regarded as easy, with a low risk of complications. None of the patients in our series had to undergo a surgical revision. Studies showed that 68% of the surgical revisions of SCS devices with non-rechargeable IPG and 75% of SCS devices with rechargeable IPG were performed in relation to the IPG alone, for example infections in the IPG area or battery replacements^1^. The lack of an IPG in this system may explain the absence of surgical revisions in our series. While problems with the implanted hardware are well known, there were a significant number of patients who experienced problems with the extracorporeal parts of the pacing system, but these can be easily replaced without invasive procedures. Manufacturers should try to improve the external component of the wSCS system to increase reliability and patient comfort.

Patients used stimulation for an average of 17 h per day demonstrating that wSCS is a suitable approach to enable active therapy not only in a periodic or intermittent manner but during the entire time the patient is awake—comparable to conventional SCS systems. This is also confirmed by the patients, as they were able to wear and use the stimulation as long as needed.

Furthermore, patients were not affected in their daily activities such as driving a car. Studies have shown that patients of advanced age are well capable of using rechargeable technologies such as smartphones and tablets^5,11^. Hence it stands to reason that age should not be a limiting factor when considering wSCS over a conventional system.

Since wSCS is a novel and innovative therapy, the number patients being eligible to assess convenience and handling is still low. Therefore, data included in the questionnaire which was designed for SCS therapy in general (independent from IPG or EPG-based systems), which is not directly related to the main points of interest, is included in the supplement.

Main limitations of our study were short-term follow-up and size of study population. Therefore, no subgroup analysis could be conducted to identify patients with certain characteristics that could best benefit from the SCS wireless system. As this innovation is quite new, we will address these uncertainties in the future with more patients and prospective studies.

In conclusion, patients felt confident using extracorporeal wSCS devices and rated the effort to maintain therapy with this system as low. The results of this pilot study need to be verified with a larger cohort in a prospective trial.

## Supplementary Information

Supplementary Information 1.

Supplementary Information 2.
