# Supplementary material for: Questionnaire-based approach to evaluate the convenience of rechargeable extracorporeal pulse generators for wireless spinal cord stimulation
Source: Sci Rep. 2022 May 17;12:8127. doi: 10.1038/s41598-022-11778-5 (PMC9113993; doi:10.1038/s41598-022-11778-5)
Supplement: Supplementary file 1 — Supplementary Information 1. [file 41598_2022_11778_MOESM1_ESM.docx]

Appendix 1 Patients ‘characteristics, Length of therapy and Patients‘ experience

*The detailed data are included in appendix 2.

| **Patients‘ characteristics** | **Number of patients (%)** |
| --- | --- |
| **Gender** |  |
| Male | 5 (83.3%) |
| Female | 1 (17.6%) |
|  |  |
| **Diagnosis** |  |
| Failed Back Surgery Syndrome | 4 (66.7%) |
| Polyneuropathy | 1 (16.7%) |
| Singultus | 1 (16.7%) |
| **Stimulation** | **Mean ± SD** |
| Duration of therapy (months) | 20.3 ± 15.9 |
|  |  |
| Stimulation per day (hours) | 17 ± 5.9 |
| **Patients‘ experience** | **Number of patients (%)** |
| **User confidence** |  |
| Confident | 5 (83.3%) |
| Not confident | 1 (16.7%) |
|  |  |
| **Overall convenience** |  |
| Easy* | 5 (83.3%) |
| Neutral | 1 (83.3%) |
|  |  |
| **Effort of the charging process and wearing of the stimulator** |  |
| Low | 3 (50.0%) |
| Neutral | 3 (50.0%) |
|  |  |
| **Active participation in therapy** |  |
| Yes* | 5 (83.3%) |
| Neutral | 1 (17.6%) |
