# Supplementary material for: Questionnaire-based approach to evaluate the convenience of rechargeable extracorporeal pulse generators for wireless spinal cord stimulation
Source: Sci Rep. 2022 May 17;12:8127. doi: 10.1038/s41598-022-11778-5 (PMC9113993; doi:10.1038/s41598-022-11778-5)
Supplement: Supplementary file 2 — Supplementary Information 2. [file 41598_2022_11778_MOESM2_ESM.docx]

Appendix 2 The additional Questionnaire with 36 questions and answers

| Question | Answers |  |
| --- | --- | --- |
| 1. Confident handling? | Yes  No | *Absolute/relative number*  5 (83.3 %)  1 (16.7 %) |
| 1. Reason for implantation of wSCS? | *Open question with free text* | - Flexibility, minimal-invasive - No sequence surgery - Recommendation, minimal-invasive - Recommendation - No answer - No answer |
| 1. Person making the decision for the wSCS System? | Patient  Doctor  Family  Nurse | *Absolute/relative number*  3 (50.0 %)  3 (50.0 %)  0  0 |
| 1. Number of instructions given? | 1 instruction  >1 instruction | *Absolute/relative number*  5 (83.3 %)  1 (16.7 %) |
| 1. Sufficiently prepared after last instruction? | Yes  No | *Absolute/relative number*  6 (100 %)  0 |
| 1. Number of stimulators? | 2  3  More than 3 | *Absolute/relative number*  4 (66.7 %)  2 (33.3 %)  0 |
| 1. Knowledge of the stimulation program? | Yes  No | *Absolute/relative number*  4 (66.7 %)  2 (33.3 %) |
| 1. Stimulation per day? | *Open question with free text* | - 24 hours - 24 hours - 10 hours - 16 hours - 16 hours - 12 hours |
| 1. Overnight use? | Yes  No | *Absolute/relative number*  2 (33.3 %)  4 (67.7 %) |
| 1. Checking charge status? | 15-30 min  1-3 hours  3-6 hours  6-12 hours | *Absolute/relative number*  1 (16.7 %)  1 (16.7 %)  1 (16.7 %)  3 (50.0 %) |
| 1. Change of stimulator per day? | *Open question with free text* | - 3 times - 6 times - 0 - 3 times - 1 time - 0 |
| 1. Charging at what battery level? | <25 %  25-50 %  50-75%  >75 %  warning signal | *Absolute/relative number*  1 (16.7 %)  0  1 (16.7 %)  0  4 (67.7 %) |
| 1. Duration of charging per stimulator? | *Open question with free text* | - 4 hours - 2 hours - 1,5 hours - 2 hours - 1 hours - 3 hours |
| 1. Pleased with time required for charging? | Yes  No | *Absolute/relative number*  6 (100.0 %)  0 |
| 1. Checking charging status of stimulator? | Patient  Family  Nurse  Other | *Absolute/relative number*  6 (100.0 %)  0  0  0 |
| 1. Charging of stimulator? | Patient  Family  Nurse  Other | *Absolute/relative number*  6 (100.0 %)  0  0  0 |
| 1. Travel during therapy? | Yes  No | *Absolute/relative number*  2 (33.3 %)  4 (67.7 %) |
| 1. Working? | Yes  No | *Absolute/relative number*  1 (16.7 %)  5 (83.3 %) |
| 1. Driving? | Yes  No | *Absolute/relative number*  5 (83.3 %)  1 (16.7 %) |
| 1. Use of electronic devices? | Yes  No | *Absolute/relative number*  5 (83.3 %)  1 (16.7 %) |
| 1. Use of opiat pain relivers? | Yes  No | *Absolute/relative number*  2 (33.3 %)  4 (66.7 %) |
| 1. Depression? | Yes  No | *Absolute/relative number*  1 (16.7 %)  5 (83.3 %) |
| 1. Anxiety disorder? | Yes  No | *Absolute/relative number*  0  6 (100.0 %) |
| 1. Event of disconnection between stimulator and transmitter? | Yes  No | *Absolute/relative number*  1 (16.7 %)  5 (83.3 %) |
| 1. Evaluation of various aspects of the neurostimulator? | Very difficult, difficult, neutral, easy, very easy | *Checking of the charging state:*  Very difficult: 1  Difficult: 0  Neutral: 2  Easy: 2  Very easy: 1  *Taking on/off the stimulator:*  *Very difficult: 0*  Difficult: 1  Neutral: 0  Easy: 3  Very easy: 2  *Charging of the stimulator:*  Very difficult: 0  Difficult: 0  Neutral: 0  Easy: 1  Very easy: 5  *Holding connection between transmitter and stimulator:*  Very difficult: 1  Difficult: 0  Neutral: 0  Easy: 3  Very easy: 2 |
| 1. Evaluation of the overall handling? | Very difficult, difficult, neutral, easy, very easy | Very difficult: 0  Difficult: 0  Neutral: 1  Easy: 5  Very easy: 0 |
| 1. Evaluation of the overall effort? | Very high, high, neutral, low, very low | Very high: 0  High: 0  Neutral: 3  Low: 3  Very low: 0 |
| 1. Evaluation of the size of the stimulator? | Very big, big, neutral, small, very small | Very big: 0  Big: 1  Neutral: 3  Small: 2  Very small: 0 |
| 1. Wearing of the stimulator as a reminder of the disease? | Completely agree, agree, neutral, disagree, completely disagree | Completely agree: 0  Agree: 2  Neutral: 2  Disagree: 0  Completely disagree: 2 |
| 1. Wearing the stimulator as active participation in therapy? | Completely agree, agree, neutral, disagree, completely disagree | Completely agree: 1  Agree: 4  Neutral: 1  Disagree: 0  Completely disagree: 0 |
| 1. Fear of forgetting to charge? | Completely agree, agree, neutral, disagree, completely disagree | Completely agree: 0  Agree: 0  Neutral: 0  Disagree: 1  Completely disagree: 5 |
| 1. Need to replace components of the system after surgery? | Yes  No | *Absolute/relative number*  *2 (33.3 %)*  *4 (66.7 %)* |
| 1. Recommendation of the stimulator? | Yes  No | *Absolute/relative number*  *5 (83.3 %)*  *1 (16.7 %)* |
| 1. Intracorporal stimulator versus wSCS? | Intracorporal  wSCS | *Absolute/relative number*  *1 (16.7 %)*  *5 (83.3 %)* |
| 1. Would choose wSCS again over conventional system? | Yes  No | *Absolute/relative number*  *5 (83.3 %)*  *1 (16.7 %)* |
| 1. Less surgery versus less charging? | Less surgery  Less charging | *Absolute/relative number*  6 (100.0 %)  *0* |
